# TPL-2 restricts Ccl24-dependent immunity to *Heligmosomoides polygyrus*

**DOI:** 10.1371/journal.ppat.1006536

**Published:** 2017-07-31

**Authors:** Yashaswini Kannan, Lewis J. Entwistle, Victoria S. Pelly, Jimena Perez-Lloret, Alan W. Walker, Steven C. Ley, Mark S. Wilson

**Affiliations:** 1 Allergy and Anti-helminth Immunity Laboratory, The Francis Crick Institute, London, United Kingdom; 2 Pathogen Genomics Group, Wellcome Trust Sanger Institute, Hinxton, Cambridgeshire, United Kingdom; 3 Microbiology Group, The Rowett Institute, University of Aberdeen, Foresterhill, Aberdeen, United Kingdom; 4 Immune Cell Signaling Laboratory, The Francis Crick Institute, United Kingdom; 5 Immunology Discovery. Genentech Inc. South San Francisco, CA, United States of America; University of Medicine & Dentistry New Jersey, UNITED STATES

## Abstract

TPL-2 (COT, MAP3K8) kinase activates the MEK1/2-ERK1/2 MAPK signaling pathway in innate immune responses following TLR, TNFR1 and IL-1R stimulation. TPL-2 contributes to type-1/Th17-mediated autoimmunity and control of intracellular pathogens. We recently demonstrated TPL-2 reduces severe airway allergy to house dust mite by negatively regulating type-2 responses. In the present study, we found that TPL-2 deficiency resulted in resistance to *Heligmosomoides polygyrus* infection, with accelerated worm expulsion, reduced fecal egg burden and reduced worm fitness. Using co-housing experiments, we found resistance to infection in TPL-2 deficient mice (*Map3k8*^*–/–*^*)* was independent of microbiota alterations in *H*. *polygyrus* infected WT and *Map3k8*^*–/–*^mice. Additionally, our data demonstrated immunity to *H*. *polygyrus* infection in TPL-2 deficient mice was not due to dysregulated type-2 immune responses. Genome-wide analysis of intestinal tissue from infected TPL-2-deficient mice identified elevated expression of genes involved in chemotaxis and homing of leukocytes and cells, including *Ccl24* and alternatively activated genes. Indeed, *Map3k8*^*–/–*^mice had a significant influx of eosinophils, neutrophils, monocytes and *Il4*^*GFP*+^ T cells. Conditional knockout experiments demonstrated that specific deletion of TPL-2 in CD11c^+^ cells, but not Villin^+^ epithelial cells, LysM^+^ myeloid cells or CD4^+^ T cells, led to accelerated resistance to *H*. *polygyrus*. In line with a central role of CD11c^+^ cells, CD11c^+^ CD11b^+^ cells isolated from TPL-2-deficient mice had elevated *Ccl24*. Finally, Ccl24 neutralization in TPL-2 deficient mice significantly decreased the expression of *Arg1*, *Retnla*, *Chil3* and *Ear11* correlating with a loss of resistance to *H*. *polygyrus*. These observations suggest that TPL-2-regulated *Ccl24* in CD11c^+^CD11b^+^ cells prevents accelerated type-2 mediated immunity to *H*. *polygyrus*. Collectively, this study identifies a previously unappreciated role for TPL-2 controlling immune responses to *H*. *polygyrus* infection by restricting Ccl24 production.

## Author summary

Helminth infections remain a huge global burden, causing significant morbidity in both animals and humans. Morbidity and recurring infections are associated with limited access to anthelmintic drugs. While vaccination remains the best available solution to treat helminthiasis, mechanisms of natural or vaccine-mediated immunity to helminths are unclear and efforts are being made to understand genetic factors and immune responses that mediate protection from infection. In this study, we tested and identified the role of a kinase, TPL-2 in regulating protective immunity to the intestinal roundworm, *Heligmosomoides polygyrus*. Using murine models with deletion of TPL-2 in all cells (*Map3k8*^*–/–*^), we identified the absence of TPL-2 protein was important for mediating protection against infection by the worm. We identified this role for TPL-2 in regulating immunity to *H*. *polygyrus* infection was not due to changes in the classical immune responses or intestinal microbiota between TPL-2 deficient and TPL-2 sufficient-wild type (WT) mice. Using genome-wide analyses and murine models of infection we discovered that TPL-2 restricted the expression of Ccl24 and the influx of innate immune cells and T cells in the small intestines of *H*. *polygyrus* infected mice. Finally we demonstrated TPL-2 mediated expression of Ccl24 is important for developing accelerated immune responses to the worm finally leading to resistance to infection by *H*. *polygyrus*. These results reveal a previously unappreciated role for TPL-2 in limiting protection to *H*. *polygyrus* infection. Thus, targeting TPL-2 could be advantageous to the development of anti-helminth therapies.

## Introduction

*Heligmosomoides polygyrus* is a natural murine intestinal helminth, used to model chronic human helminth infections. Resistance to *H*. *polygyrus* is mediated by genetic strain specific responses [1], as well as protective immune mechanisms attributed to the strength of the type-2 immune response [2]. These include the activation of alternatively activated macrophages leading to the killing of tissue dwelling larvae [3], production of IgG1 antibodies that limit parasite fecundity and protect against reinfection [4, 5], and production of the anti-parasitic protein RELM-β by intestinal epithelial cells [6]. Despite these mechanistic observations of resistance to infection by *H*. *polygyrus*, there remains a considerable gap in our understanding of the molecular pathways regulating natural or vaccine mediated immunity to *H*. *polygyrus*.

Tumor Progression Locus 2 (TPL-2; also known as MAP3K8 and COT), activates the ERK1/2 MAP kinase pathway in response to stimulation of Toll-like receptors (TLRs), TNF receptor (TNFR) and IL-1 (IL-1R) [7]. TPL-2 is an important kinase for pro-inflammatory type-1 and Th17 responses [8, 9]. TPL-2/ERK1/2 signaling is required for cytokine and chemokine production, with TPL-2-deficient (*Map3k8*^–/–^) mice being protected in models of endotoxic shock, experimental autoimmune encephalomyelitis (EAE) [9], pancreatitis, liver fibrosis and thrombocytopenia [10–15]. Additionally, TPL-2 is required for protective immunity to intracellular bacterial and protozoan infection, with substantially greater susceptibility in *Map3k8*^–/–^mice compared to wild type [8, 16].

In contrast to these pro-inflammatory roles for TPL-2 in type-1/Th17 responses, we recently identified a regulatory role for TPL-2 in type-2 immune settings, including house dust mite (HDM) mediated airway allergy. *Map3k8*^–/–^mice demonstrated significantly increased production of IL-4, IL-5 and IL-13, increased airway eosinophilia, severe lung inflammation and increased serum IgE compared to WT mice [17]. TPL-2 also regulated alternative activation of macrophages, with *Map3k8*^–/–^mice developing significantly greater fibrotic granulomas with enhanced type-2 immune responses following *Schistosoma mansoni* infection [18]. While, increased type-2 responses contributed to increased immunopathology following HDM allergen challenge or *S*. *mansoni* infection, in this study we tested the hypothesis that TPL-2 regulated type-2 immune responses contributed to susceptibility to intestinal helminth infection.

In the present study, we demonstrate that *Map3k8*^–/–^mice were resistant to a primary *H*. *polygyrus* infection, with significantly fewer worm and fecal egg burdens compared to wild type (WT) infected mice. Resistance to *H*. *polygyrus* in *Map3k8*^*–/–*^mice was not due to differences in type-2 immune responses with comparable adaptive and cytokine responses. Given the importance of intestinal microbiota in regulating local and systemic immune responses [19] and the fact that TPL-2 is downstream of TLR-4 [10], we investigated the impact on intestinal microbial communities following *H*. *polygyrus* infection of WT and *Map3k8*^–/–^mice. Although intestinal microbiota composition was altered following infection and that distinct differences were observed in *Map3k8*^–/–^mice, co-housing experiments that corrected for these between-genotype variations ruled out any role for microbiota alterations in mediating protective immunity to *H*. *polygyrus* in *Map3k8*^–/–^mice. Transcriptional analysis of intestinal tissue from infected mice revealed increased expression of *Ccl24* alongside a significant increase in the expression of type-2 memory signature genes associated with alternatively activated cells, including *Arg1*, *Chil3* and *Ear11* in *Map3k8*^–/–^mice, compared to WT mice. Using lineage specific conditional knockout (KO) mice, we identified a role for TPL-2 in CD11c^+^ cells and not Villin^+^ epithelial cells, LysM^+^ myeloid cells or CD4^+^ T cells in controlling susceptibility to *H*. *polygyrus*. CD11c^+^ CD11b^+^ cells from *H*. *polygyrus* infected *Map3k8*^–/–^mice expressed increased *Ccl24* compared to WT mice. Increased *Ccl24* expression correlated with an increase in the frequency of eosinophils, neutrophils, monocytes and *Il4*^*GFP+*^ Th2 cells in *Map3k8*^–/–^mice, compared to WT mice. Furthermore, blocking Ccl24 *in vivo* resulted in a significant decrease in the expression of type-2 memory markers, *Arg1*, *Retnla*, *Chil3* and *Ear11* and led to loss of resistance to *H*. *polygyrus* in *Map3k8*^–/–^mice. These data suggest that TPL-2-regulated Ccl24 is an important pathway contributing to susceptibility to *H*. *polygyrus*. These results demonstrate a previously unidentified role for TPL-2 in restricting protective Ccl24-dependent pathways during intestinal *H*. *polygyrus* infection.

## Results

### *Map3k8*^*–/–*^mice are resistant to *Heligmosomoides polygyrus* infection

**Fig 1:**
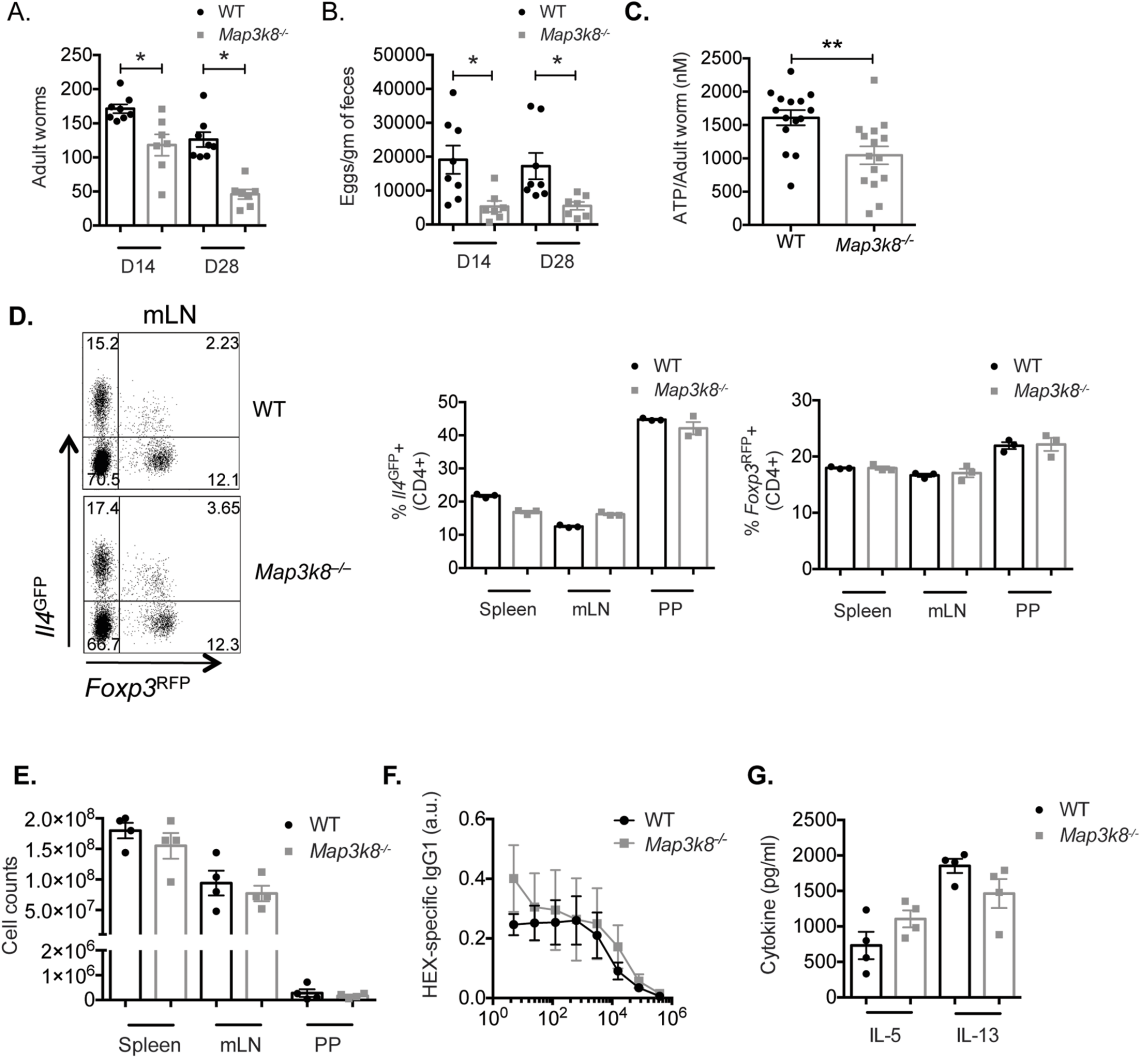
*Map3k8*^*–/–*^mice are resistant to *H*. *polygyrus* infection. A) WT and *Map3k8*^*–/–*^mice were infected with 200 L3 stage *H*. *polygyrus* larvae. Adult luminal worms from the intestinal tissue were counted on days 14 (D14) and 28 (D28). B) Fecal egg burden in *H*. *polygyrus* infected WT and *Map3k8*^*–/–*^mice at D14 and D28. C) ATP levels of adult *H*. *polygyrus* worms harvested from the duodenal tissue of WT and *Map3k8*^*–/–*^mice at day 14 post infection. D) Representative FACS plots from the mesenteric lymph nodes (mLNs) and frequency of CD4^+^ *Il4*^GFP+^ and CD4^+^ *FoxP3*^RFP+^ T cells from the spleen, mLNs and Peyer’s patches (PP) of D14 *H*. *polygyrus* infected WT and *Map3k8*^*–/–*^mice. E) Total number of cells from the spleen, mLNs and PP of D14 *H*. *polygyrus* infected WT and *Map3k8*^*–/–*^mice. F) Arbitrary units (a.u.) of *H*. *polygyrus* adult worm extract (HEX)-specific IgG1 in the serum of D14 *H*. *polygyrus* infected WT and *Map3k8*^*–/–*^mice. G) HEX-specific IL-5 and IL-13 in mLN cell culture supernatants of D14 *H*. *polygyrus* infected WT and *Map3k8*^*–/–*^mice. Data from readouts in A) and B) represent 7–8 mice/genotype pooled from 2 independent experiments. Data from C)—G) is representative of 2–3 independent experiments with 3–4 mice/genotype and 4 adult worms/mouse. * and ** denote p≤0.05 using a two-tailed Mann-Whitney test.

To test whether TPL-2 contributed to immunity to *H*. *polygyrus*, we infected WT and *Map3k8*^*–/–*^mice with 200 *H*. *polygyrus* L3 stage larvae. Adult luminal worms and fecal eggs were evaluated on day 14 (D14) and D28 post infection. *Map3k8*^*–/–*^mice harbored significantly fewer worms at D14 compared to WT mice, with a further significant reduction in worm burden by D28 (**Fig 1A**). Parasite egg burden in *Map3k8*^*–/–*^mice was also significantly reduced compared to WT mice at D14 and D28 (**Fig 1B**). To test whether the deficiency of TPL-2 in the host directly affected the fitness of the remaining worms, we measured the level of ATP [6] in the remaining adult worms harvested from the lumen of D14 *H*. *polygyrus* infected WT and *Map3k8*^*–/–*^mice. The worms harvested from the lumen of *Map3k8*^*–/–*^mice had significantly lower ATP levels compared to WT mice (**Fig 1C**), suggesting that the absence of TPL-2 led to lower worm burden likely by compromising the fitness of *H*. *polygyrus*.

It has previously been suggested that T cell intrinsic TPL-2 regulates Th2 differentiation [20] and regulatory T cell development [21]. IL-4^+^ Th2 cells are important for generating a protective type-2 response in *H*. *polygyrus* infection [22, 23] and *Foxp3*-expressing regulatory T cells (Tregs) are important for regulating Th2 immune responses during *H*. *polygyrus* infection [24]. Therefore, to determine if Th2 and Treg frequencies and numbers were affected in WT and *Map3k8*^*–/–*^mice following *H*. *polygyrus* infection, we crossed *Map3k8*^*–/–*^mice onto an *Il4*^GFP^*Foxp3*^RFP^ dual-reporter background to simultaneously monitor Th2 and Treg cells during *H*. *polygyrus* infection. Analysis of Th2 cells in the spleen, mesenteric lymph node (mLN) or Peyer’s patches (PP) revealed there was no significant difference in the frequency of *Il4*^GFP+^ CD4^+^ Th2 cells between infected WT and *Map3k8*^*–/–*^mice at D14 (**Fig 1D**). Similarly, we found no significant difference in the frequency of *Foxp3*^RFP+^ Treg cells between the mice at D14 post infection (**Fig 1D**). Of note, there was no significant difference in the total cell numbers among the different tissues between WT and *Map3k8*^*–/–*^mice (**Fig 1E**).

Anti-parasitic immunity has been correlated with serum IgG1 [5]. We therefore measured *H*. *polygyus* antigen extract (HEX)-specific IgG1 in the serum of infected mice. Both WT and *Map3k8*^*–/–*^mice mounted significant, but indistinguishable amounts of HEX-specific IgG1 (**Fig 1F**). Similarly, there were no significant differences in the production of HEX specific type-2 cytokines, IL-5 and IL-13 in mLN cell culture supernatants from infected WT and *Map3k8*^*–/–*^mice (**Fig 1G**). These data indicated that TPL-2 deficiency promoted immunity to *H*. *polygyrus*, but this is not associated with any change in T cell-associated responses.

### Anti-helminth immunity in *Map3k8*^*–/–*^mice was independent of intestinal microbiota composition.

**Fig 2:**
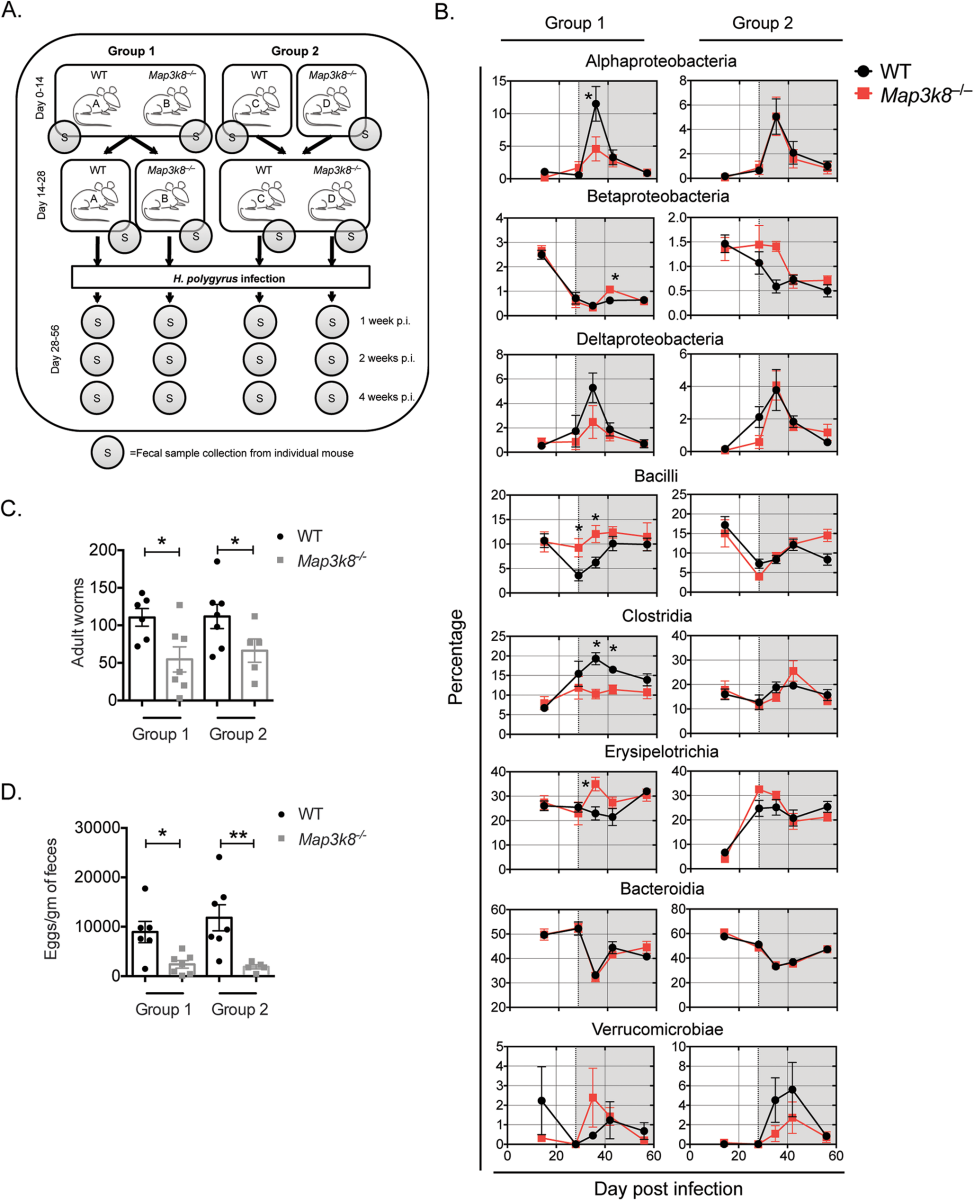
Accelerated expulsion in *Map3k8*^*–/–*^mice not due to alterations in the intestinal microbiota. A) Schematic representation of co-housing, infection and fecal sample collection from two different groups of WT and *Map3k8*^*–/–*^mice. Group 1 mice were co-housed together for 14 days and then separated into the different genotypes for the next 14 days. Group 2 mice included the two genotypes housed individually for 14 days following which they were cohoused together for 14 days. Both groups of mice were infected with 200 L3 stage *H*. *polygyrus* larvae and fecal samples (S) were collected at the indicated times points. B) Changes in fecal microbiota composition over time (represented as a percentage of total bacterial content) in the two different groups of WT and *Map3k8*^*–/–*^mice infected with *H*. *polygyrus*. Data represents 5 mice/genotype. Shaded grey sections indicate the time points after infection with *H*. *polygyrus*. C) Adult luminal worm burden in the two different groups of WT and *Map3k8*^*–/–*^mice infected with *H*. *polygyrus* at day 28. D) Fecal egg burden in the two different groups of WT and *Map3k8*^*–/–*^mice infected with *H*. *polygyrus* at day 28. Data represents 6 mice/group pooled from 2 independent experiments. * denotes p≤0.05 using Mann-Whitney test.

A close relationship between helminth infections and intestinal microbiota has previously been reported [25], with a recent study demonstrating a direct correlation between abundance of *Lactobacillus* species and susceptibility to infection by *H*. *polygyrus* [26]. To evaluate whether changes in intestinal microbiota contribute to resistance in *Map3k8*^*–/–*^mice, we setup a co-housing experiment between WT and *Map3k8*^*–/–*^mice as depicted in the schematic (**Fig 2A**). WT and *Map3k8*^*–/–*^mice were co-housed in two groups. Group 1 allowed for genotype-specific influences on microbiota to emerge, while group 2 aimed to normalize any impact on microbiota.

Briefly, group 1 mice were co-housed together for 14 days, normalizing the microbiota between all mice and then separated into their individual genotypes for 14 days for genotype associated differences in microbiota to emerge. For the remainder of the experiment, group 1 mice were left in their genotype-associated separate housing. Group 2 mice were kept separated for 14 days before being co-housed together for 14 days. For the remainder of the experiment, mice in group 2 were co-housed. Fecal pellets were collected on day 14 and day 28 (as depicted by “S” in Fig 2B).

Both groups of mice were subsequently infected with *H*. *polygyrus* on D28 and fecal samples were harvested at 1 (day 35), 2 (day 42) and 4 (day 56) weeks-post infection, as indicated. Analysis of the fecal microbiota composition by 16S ribosomal RNA sequencing revealed no significant differences in the fecal microbiota of the WT and *Map3k8*^*–/–*^mice at day 14 of the experimental setup (**Fig 2B**). However, a genotype-associated influence on *Bacilli* spp. emerged in group 1 at day 28, with a significant proportional increase in *Map3k8*^*–/–*^mice compared to WT mice, which was further increased following 1 week (day 35) of *H*. *polygyrus* infection. Conversely, *Alphaproteobacteria* spp., *Clostridia* spp. and *Erysipelotrichia* spp. were proportionally increased in WT mice compared to *Map3k8*^*–/–*^mice following 1-week of *H*. *polygyrus* infection (**Fig 2B**). This time point corresponds with larval migration through the mucosal surface of the small intestine. These differences correlated with an increase in overall microbial diversity in WT, but not *Map3k8*^*–/–*^, mice (**S2 Fig**). At 2-weeks (day 42) post-infection, there was a secondary shift in the microbial composition, with a significant proportional increase in *Betaproteabacteria* spp. and a relative reduction in *Clostridia* spp. in *Map3k8*^*–/–*^mice (**Fig 2B**). Correlating with the changes in microbial composition described in group 1, *Map3k8*^*–/–*^mice expelled significantly more worms (**Fig 2C**) with significantly lower eggs detected in the feces (**Fig 2D**), similar to previous observations (**Fig 1**). In group 2, co-housing mice normalized all of the above-mentioned differences between the genotypes following infection, indicating that the co-housing setup had successfully corrected for genotype-associated influence on microbial communities. Normalization of microbiota between WT and *Map3k8*^*–/–*^mice had no impact on the observed resistance in *Map3k8*^*–/–*^mice, with fewer worms (**Fig 2C**) and fewer eggs detected in fecal pellets of *Map3k8*^*–/–*^mice (**Fig 2D**). These data indicated that there were changes in microbial composition following *H*. *polygyrus* infection and that these changes were regulated, in part, by TPL-2. However, changes in microbial composition in *Map3k8*^*–/–*^mice did not contribute to the resistance phenotype observed.

### Resistance to *H*. *polygyrus* infection in *Map3k8*^*–/–*^mice was not due to differential Th2 priming

Following the observation that fewer adult worms were present at D14 (**Fig 1A**), we assessed the early type-2 immune response at D5 and D7-post infection to assess early Th2 priming events. No differences in cellularity, or the frequency of CD4^+^ *Il4*^GFP+^ Th2 cells or *Foxp3*^RFP+^ CD4^+^ cells were observed in the spleen, mLN or PP between infected WT and *Map3k8*^*–/–*^mice at D5 or D7 (**S1A–S1F Fig**). Similarly, HEX-specific IL-5 and IL-13 production from mLN cell culture supernatants of infected WT and *Map3k8*^*–/–*^mice were similar at D7 (**S1G Fig**). These observations suggested that resistance to infection in *Map3k8*^*–/–*^mice was not due to dysregulated Th2 cell priming. Following infection, infective larvae penetrate the mucosal layer and embed in the external muscularis layer of the duodenum [1]. To determine whether a similar number of larvae were infecting and penetrating through the mucosal layer, we evaluated number of *H*. *polygyrus* larvae in the duodenal wall at D5 post infection. Significantly fewer worms were present in the intestinal wall of *Map3k8*^*–/–*^mice, compared to WT mice (**S1H Fig**). These results indicated an early event in the tissue might impact the emergence and fitness of *H*. *polygyrus* leading to increased resistance in *Map3k8*^*–/–*^mice.

### Increased expression of *Ccl24* and genes associated with type-2 memory responses in *Map3k8*^*–/–*^mice

**Fig 3:**
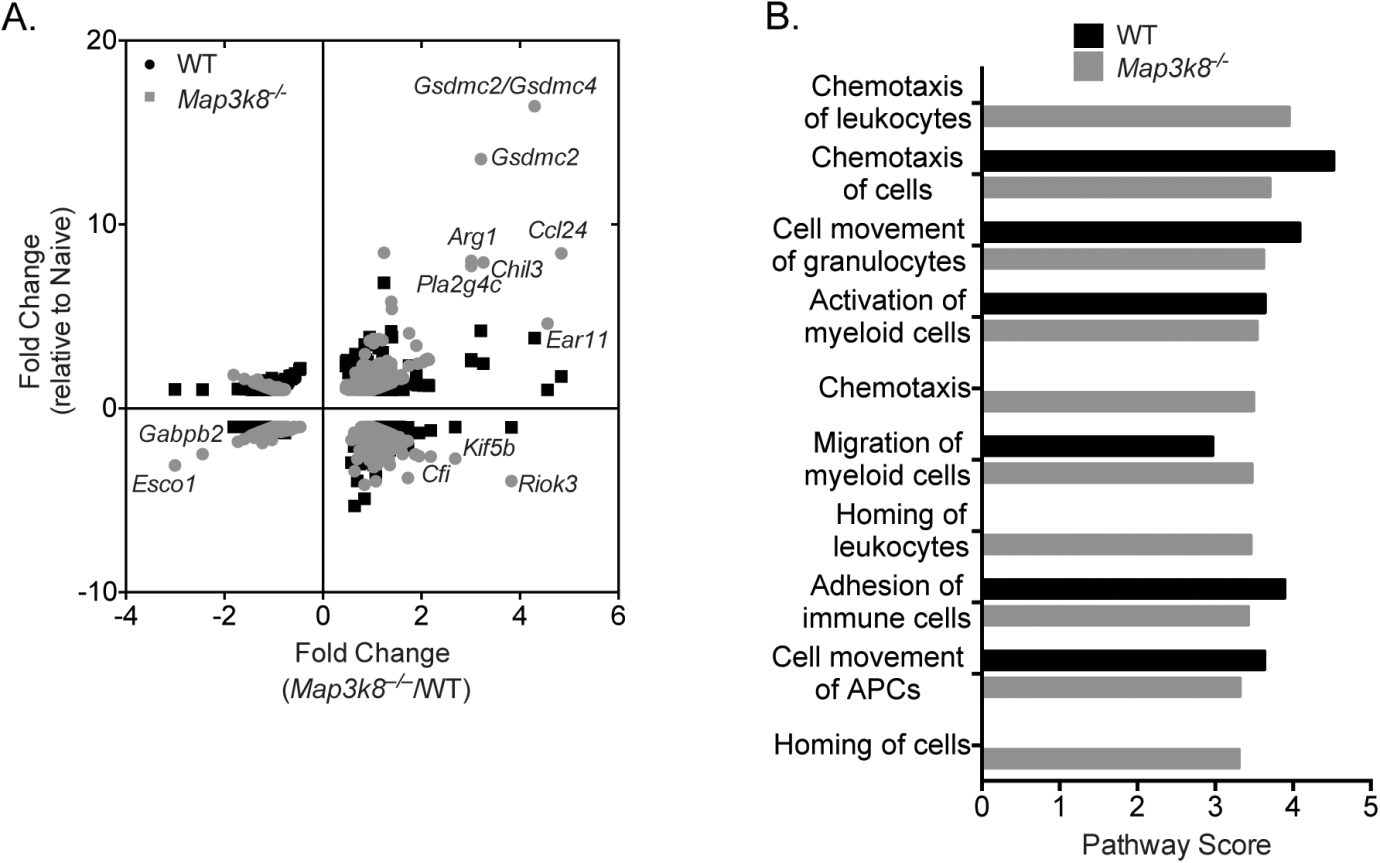
Elevated Ccl24 from *Map3k8*^*–/–*^mice mediates resistance to *H*. *polygyrus* infection. A) Ratios of ratio plot depicting differentially expressed genes in the duodenal tissue of D5 *H*. *polygyrus* infected WT and *Map3k8*^*–/–*^mice. The x axis represents fold change for gene differentially expressed in infected *Map3k8*^*–/–*^mice over infected WT mice and the y axis represents fold change for gene differentially expressed in infected WT or *Map3k8*^*–/–*^mice relative to their respective naïve uninfected controls. B) Top 10 gene regulatory pathways upregulated in D5 *H*. *polygyrus* infected *Map3k8*^*–/–*^/ WT mice gut tissue relative to naive control mice as predicted by ingenuity pathway analysis (IPA). The microarray analysis was performed on duodenal tissue from 3 biological replicate samples/genotype for each group.

To identify gene expression differences that might contribute to the reduced larval establishment in *Map3k8*^*–/–*^mice, we isolated RNA from duodenal tissue at D5-post *H*. *polygyrus* infection of WT or *Map3k8*^*–/–*^mice. Transcripts in infected WT and *Map3k8*^*–/–*^mice were compared to their respective naïve controls, generating a fold-change from naïve value (**Fig 3A**, y-axis). These fold change values were then compared to each other (**Fig 3A**, x-axis) creating a ratio of ratios plot to identify genes that were significantly different in *Map3k8*^*–/–*^mice at D5-post infection (x-axis)(**Fig 3A**). This analysis identified several genes that were significantly upregulated in infected *Map3k8*^*–/–*^mice relative to infected WT mice, including *Ccl24*, *Ear11*, *Gsdmc2/Gsdmc4*, *Riok3*, *Chil3*, *Gsdmc2*, *Pla2g4c*, *Arg1*, *Kif5b and Cfi* (**Fig 3A and Table 1**). Pathway analysis identified increased chemotaxis of leukocytes, chemotaxis, homing of leukocytes and homing of cells in *Map3k8*^*–/–*^mice, which were completely absent in infected WT mice (**Fig 3B**). These observations suggested that an early upregulation of genes/pathways involved in chemotaxis and type-2 memory responses might be responsible for the enhanced resistance observed in *Map3k8*^*–/–*^mice.

**Table 1:** Top 10 upregulated genes in D5 *H*. *polygyrus* infected *Map3k8*^*–/–*^mice relative to WT mice.

Gene	Fold Change (*Map3k8*^*–/–*^/ WT)
*Ccl24*	4.843
*Ear11*	4.560
*Gsdmc2/Gsdmc4*	4.297
*Riok3*	3.828
*Chil3*	3.259
*Gsdmc2*	3.207
*Pla2g4c*	3.011
*Arg1*	3.010
*Kif5b*	2.686
*Cfi*	2.190

Fold change of the 10 highest expressed genes identified by microarray analysis in the duodenal tissue of D5 *H*. *polygyrus* infected *Map3k8*^*–/–*^mice relative to WT mice was tabulated in the order of decreasing fold change.

### *Map3k8* deficiency in CD11c^+^ cells mediates protective immunity to *H*. *polygyrus* infection

**Fig 4:**
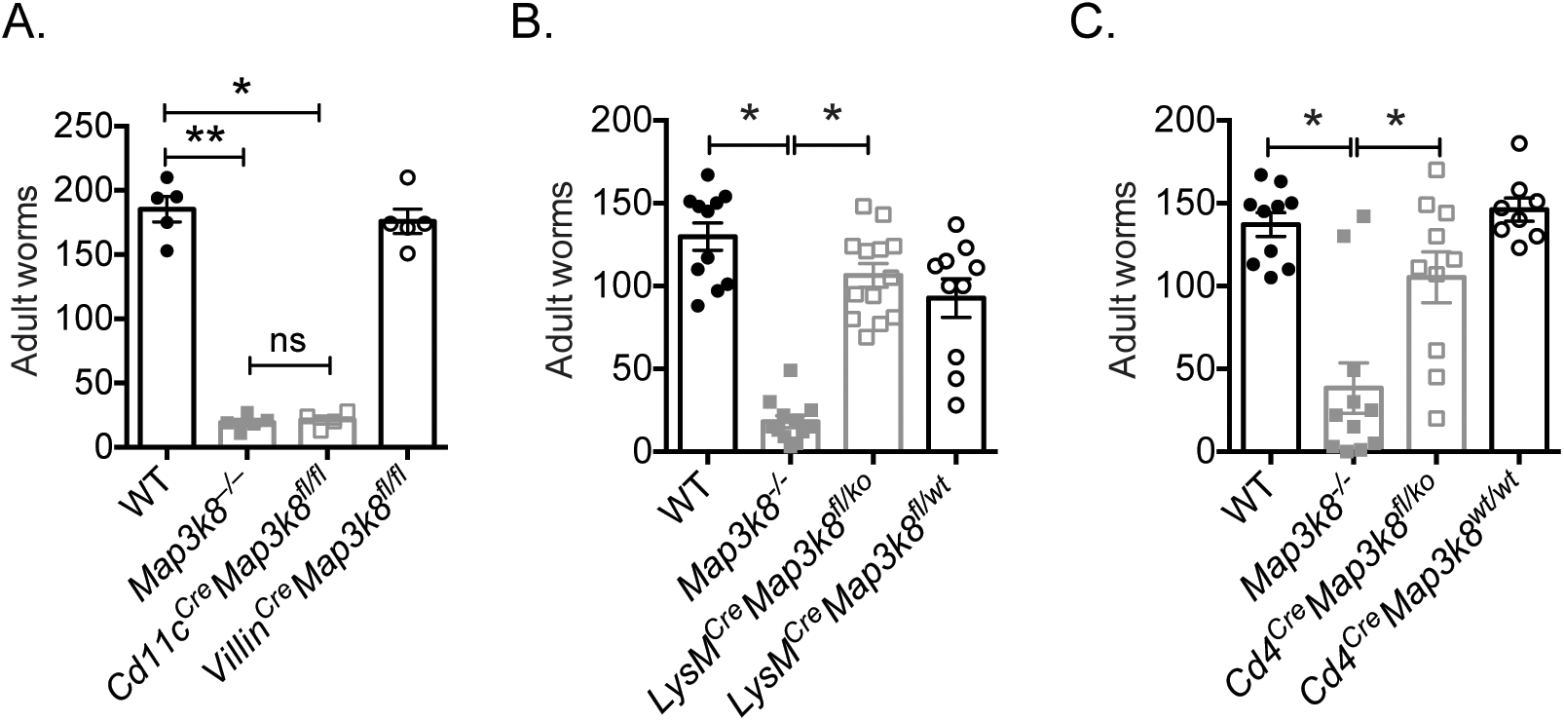
CD11c^+^ cells deficient in TPL-2 but not Villin^+^ cells, LysM^+^ cells or CD4^+^ cells mediate enhanced expulsion of *H*. *polygyrus*. A) Adult luminal worm burden in D21 *H*. *polygyrus* infected WT, *Map3k8*^*–/–*^, *Villin*^*Cre*^*Map3k8*^*fl/fl*^ and *Cd11c*^*Cre*^*Map3k8*^*fl/ko*^ mice. B) Adult luminal worm burden in D21 *H*. *polygyrus* infected WT, *Map3k8*^*–/–*^, *LysM*^*Cre*^*Map3k8*^*fl/wt*^ and *LysM*^*Cre*^*Map3k8*^*fl/ko*^ mice. C) Adult luminal worm burden in D21 *H*. *polygyrus* infected WT, *Map3k8*^*–/–*^, *Cd4*^*Cre*^*Map3k8*^*wt/wt*^ and *Cd4*^*Cre*^*Map3k8*^*fl/ko*^ mice. Data from A) is representative of 2 independent experiments with 4–5 mice/genotype and data from B) and C) is 8–13 mice/genotype pooled from 2 independent experiments. * and ** denotes p≤0.05 using Mann-Whitney test.

Next, to determine which cellular compartment was responsible for mediating resistance to *H*. *polygyrus* in *Map3k8*^*–/–*^mice, we restricted TPL-2 deficiency to cells primarily responsible for expressing the genes upregulated in D5 *H*. *polygyrus* infected *Map3k8*^*–/–*^mice (Table 1). These included CD11c^+^ cells, which have been reported to express *Ccl24* [27, 28], LysM^+^ myeloid cells/macrophages, which express *Ccl24*, *Ear11*, *Arg1* and *Chil3* [27–29] and Villin^+^ epithelial cells, which have been shown to express *Gsdmc2/ Gsdmc4* [6, 30]. To determine the role of TPL-2 in these cells *in vivo*, we assessed the adult worm burden in *Cd11c*^*Cre*^*Map3k8*^*fl/fl*^, *Villin*^*Cre*^*Map3k8*^*fl/fl*^ and *LysM*^*Cre*^*Map3k8*^*fl/ko*^ conditional knockout mice. We also evaluated the adult worm burdens in *Cd4*^*Cre*^*Map3k8*^*fl/ko*^ mice to formally test whether TPL-2 had a T cell-intrinsic role in mediating resistance to *H*. *polygyrus*. Significantly fewer worms were observed in *Cd11c*^*Cre*^*Map3k8*^*fl/fl*^ mice, comparable to *Map3k8*^*–/–*^mice, but not in *Villin*^*Cre*^*Map3k8*^*fl/fl*^ (**Fig 4A**), *LysM*^*Cre*^*Map3k8*^*fl/ko*^ or *Cd4*^*Cre*^*Map3k8*^*fl/ko*^ mice (**Fig 4B and 4C**). This suggested that a CD11c^+^ cell-intrinsic function of TPL-2 regulated resistance to *H*. *polygyrus* infection.

### Increased *Ccl24* expression in CD11c^+^ cells and a concurrent early type-2 memory-like response observed in D5 *H*. *polygyrus* infected *Map3k8*^*–/–*^mice

**Fig 5:**
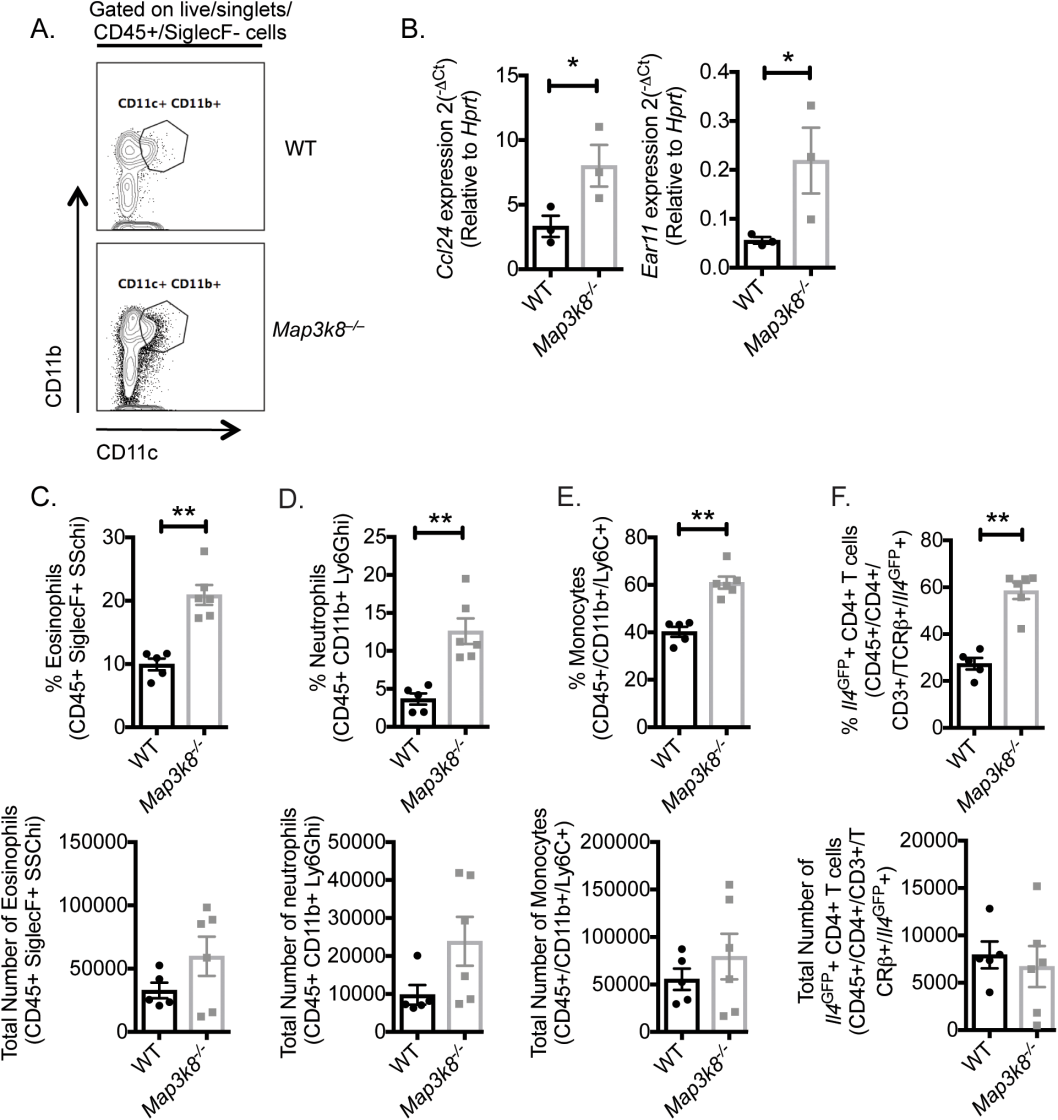
Enhanced *Ccl24*, myeloid cells and *Il4*^*GFP+*^ Th2 cell infiltration in *Map3k8*^*–/–*^mice. A) Representative FACS profile for CD11c^+^ CD11b^+^ cells from the intestinal LP of D5 *H*. *polygyrus* infected WT and *Map3k8*^*–/–*^mice. B) *Ccl24* and *Ear11* expression from CD11c^+^ CD11b^+^ cells D5 *H*. *polygyrus* infected WT and *Map3k8*^*–/–*^mice. C-H) Frequency and number of (C) eosinophils (live/CD45^+^/SiglecF^+^/SSChi); (D) neutrophils (live/CD45^+^/SiglecF^-^/CD11b^+^/Ly6G^+^); (E) monocytes (live/CD45^+^/SiglecF^-^/CD11b^+^/Ly6G^-^/Ly6C^+^); (F) *Il4*^*GFP+*^ Th2 cells (live/CD45^+^/CD4^+^/CD3^+^/TCRβ^+^/*Il4*^*GFP+*^) in the intestinal LP of D5 *H*. *polygyrus* infected WT and *Map3k8*^*–/–*^mice. Data from B) is representative of 2 independent experiments with 3 biological replicates per group. Data from C-H) is representative of 2 independent experiments with 5–6 mice/group. * and ** denotes p≤0.05 using Mann-Whitney test.

*Ccl24* was expressed in both *H*. *polygyrus* infected WT and *Map3k8*^*–/–*^mice, but was upregulated ~5.0 fold more in *Map3k8*^*–/–*^mice compared to WT mice (**Table 1)**. Ccl24 is a chemotactic factor for eosinophils, granulocytes and lymphocytes [31, 32]. Following the observation that CD11c^+^ cells deficient in *Map3k8* mediate resistance to *H*. *polygyrus* infection (**Fig 4**) and the recent observation that elevated *Ccl24* from CD11c^+^ MHC-II^+^ cells in *Map3k8*^*–/–*^mice contributed to enhanced HDM-driven airway allergy [17], we hypothesized that elevated Ccl24 in *Map3k8*^*–/–*^mice could be responsible for accelerated resistance to *H*. *polygyrus*. We therefore determined the expression of *Ccl24* in CD11c^+^ cells from the lamina propria of the duodenum and jejenum of *H*. *polygyrus* infected WT and *Map3k8*^*–/–*^mice (**Fig 5A**). CD11c^+^ CD11b^+^ cells isolated from D5 *H*. *polygyrus* infected *Map3k8*^*–/–*^mice expressed significantly increased *Ccl24* compared to similar cells from infected WT mice (**Fig 5B**). In addition, CD11c^+^ CD11b^+^ cells from *H*. *polygyrus* infected *Map3k8*^*–/–*^mice also expressed significantly higher levels of *Ear11* (**Fig 5B**), *Retnla* (**S3A Fig**), and had a trend for an increase in the expression of *Arg1* and *Chil3* (**S3B and S3C Fig**) compared to WT cells.

To determine whether the increased expression of *Ccl24* in CD11c^+^ CD11b^+^ cells was associated with enhanced cellular influx of CCR3 expressing cells, including eosinophils, granulocytes and lymphocytes [31–33], we evaluated the myeloid and lymphoid cell infiltration in the small intestinal lamina propria (LP) of D5 *H*. *polygyrus* infected *Map3k8*^*–/–*^and WT mice. Concurrent with increased *Ccl24*, we observed a significant increase in the frequency of eosinophils (**Fig 5C**), neutrophils (**Fig 5D**) and Ly6C^+^ monocytes (**Fig 5E**) in D5 *H*. *polygyrus* infected *Map3k8*^*–/–*^mice compared to WT mice. This increase in frequency was accompanied with a trend for an increase in the number of these cells in *Map3k8*^*–/–*^mice compared to WT mice (**Fig 5C–5E**). We also observed a significant increase in the frequency of *Il4*^***GFP*+**^ Th2 cells in the LP of D5 *H*. *polygyrus* infected *Map3k8*^*–/–*^mice compared to WT mice (**Fig 5F**). There was no change in the total number of *Il4*^***GFP*+**^ Th2 cells in *H*. *polygyrus* infected *Map3k8*^*–/–*^mice compared to WT mice (**Fig 5F)** most likely due to a decrease in lymphocytes (CD4^+^ T cells, CD8^+^ T cells and trend for a decrease in CD19^+^ B cells) in *Map3k8*^*–/–*^mice compared to WT mice (**S3D–S3F Fig**). There was a trend for a decreased macrophages (CD11b^+^ F4-80^+^) in *Map3k8*^*–/–*^mice compared to WT mice (**S3G Fig**), however this failed to reach statistical significance. Similarly, here was a trend for a reduced frequency of KLRG1^+^ Sca1^+^ group 2 innate lymphoid cells (ILC2) (**S3H Fig**), which also failed to reach statistical significance. The total numbers of ILC2s were unchanged.

### Ccl24 neutralization reduced early type-2 memory responses and led to decreased resistance to *H*. *polygyrus* in *Map3k8*^*–/–*^mice

**Fig 6:**
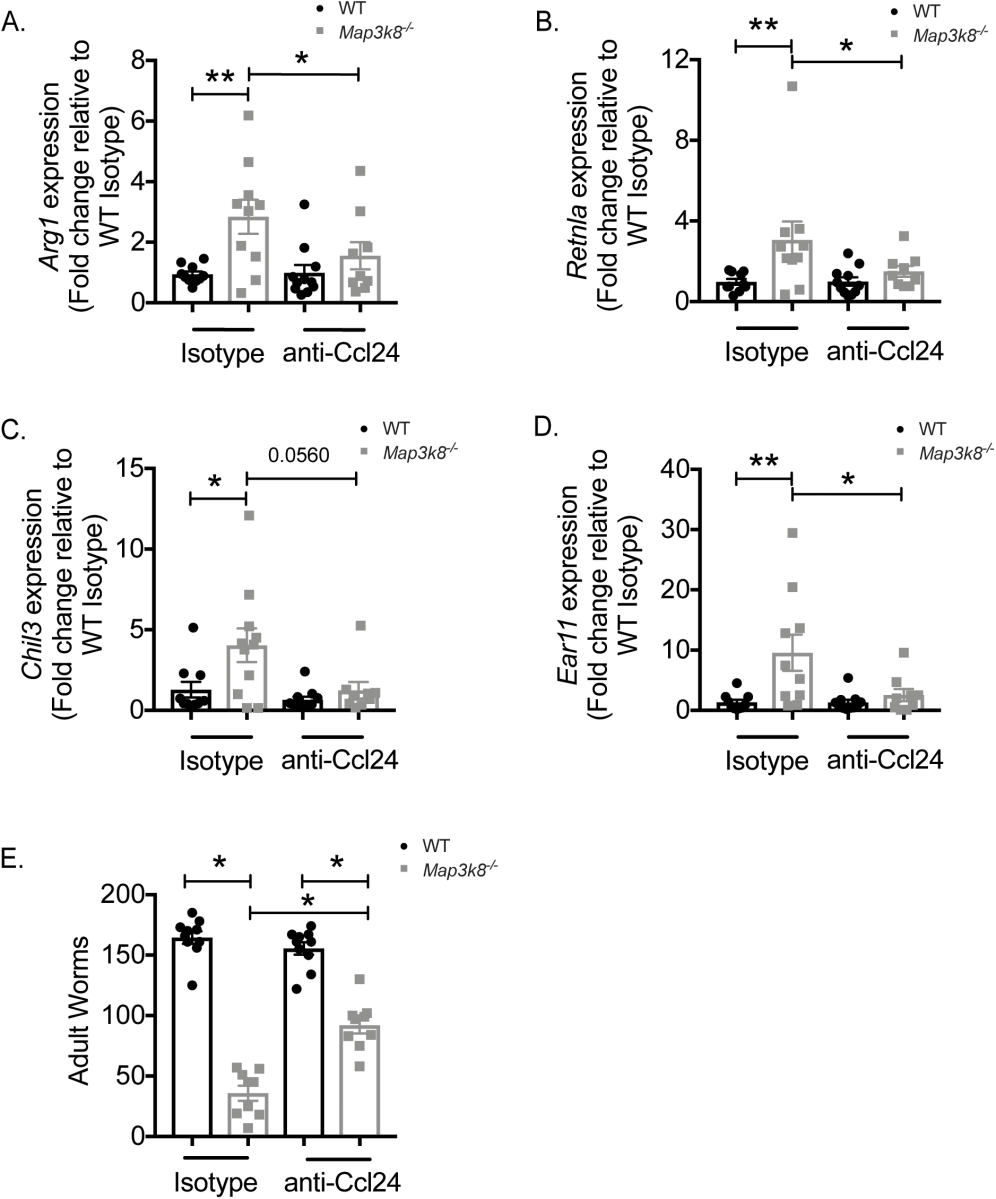
Ccl24 neutralization reduced expression of early type-2 memory responses, correlating with a loss of resistance to *H*. *polygyrus* in *Map3k8*^*–/–*^mice. A) *Arg1* expression; B) *Retnla* expression; C) *Chil3* expression; D) *Ear11* expression from small intestinal duodenal tissue of D5 *H*. *polygyrus* infected WT and *Map3k8*^*–/–*^mice treated with Ccl24 neutralizing antibody or isotype control antibody. E) Adult luminal worms in the D21 *H*. *polygyrus* infected WT and *Map3k8*^*–/–*^mice treated with Ccl24 neutralizing antibody or isotype control. Data represents 8–11 mice/genotype/group, pooled from 2 independent experiments. * denotes p≤0.05 using Mann-Whitney test.

Finally, to determine whether elevated *Ccl24* in *Map3k8*^*–/–*^mice contributed to enhanced type-2 memory responses and resistance to *H*. *polygyrus*, we neutralized Ccl24 using anti-Ccl24 antibodies in *H*. *polygyrus* infected WT and *Map3k8*^*–/–*^mice. In accordance with previous observations (**Fig 3, Table 1**) *Map3k8*^*–/–*^mice had significantly increased expression of signature genes associated with alternatively activated macrophages [29] and helminth associated type-2 memory responses [3, 34], including *Arg1* (**Fig 6A**), *Retnla* (**Fig 6B**), *Chil3* (**Fig 6C**) and *Ear11* (**Fig 6D**). Neutralization of Ccl24 led to a significant decrease in the expression of most of these type-2 memory markers in *Map3k8*^*–/–*^mice, compared to *Map3k8*^*–/–*^mice treated with control antibodies (**Fig 6A–6D**). Ccl24 blockade had no observable effect in WT mice. Similarly, neutralizing Ccl24 had no significant effect on the luminal worm burden in infected WT mice compared to isotype treated WT control mice (**Fig 6E**). However, Ccl24 blockade significantly reversed the resistance phenotype in infected *Map3k8*^*–/–*^mice, compared to isotype treated *Map3k8*^*–/–*^mice (**Fig 6E**), correlating with the loss of type-2 memory markers (Fig 6A–6D). These observations indicated that immunity to *H*. *polygyrus* in *Map3k8*^*–/–*^mice were determined, in part, by increased Ccl24, correlating with accelerated expression of type-2 memory responses.

## Discussion

Soil transmitted helminth infections remain a significant global burden, especially in tropical and subtropical countries. Anthelmintic drugs remain the only treatment option to control helminthiasis. At present the immune mechanisms regulating natural resistance to infection, and thus a putative vaccine-driven immune pathway capable of eliciting protective immunity, are still unclear. In the present study we demonstrated that TPL-2 deficiency accelerates resistance to *H*. *polygyrus* infection. Using transcriptional analysis, we identified that *Ccl24* was upregulated almost 5-fold more in infected *Map3k8*^*–/–*^mice, compared to WT mice. We also observed a significant increase in genes associated with alternatively activated macrophages and helminth associated type-2 memory responses, including *Arg1* (3-fold), *Chil3* (3.2-fold) *and Ear11* (4.5-fold), in infected *Map3k8*^*–/–*^mice, compared to WT mice. Similar to our previous observations [17], we observed that TPL-2 functions in a cell intrinsic manner in CD11c^+^ cells and that CD11c^+^ CD11b^+^ cells from D5 *H*. *polygyrus* infected mice expressed elevated *Ccl24*. Consistent with the hypothesis that Ccl24 is an important chemotactic mediator for eosinophils, granulocytes and lymphocytes, we observed a significant increase in eosinophils, neutrophils, monocytes and *Il4*^*GFP*^ expressing CD4^+^ T cells in the LP of D5 *H*. *polygyrus* infected *Map3k8*^*–/–*^mice compared to WT mice. Finally neutralizing Ccl24 led to a significant reduction in the expression of *Arg1*, *Retnla*, *Chil3* and *Ear11*, correlating with a loss of resistance in *H*. *polygyrus* infected *Map3k8*^*–/–*^mice. In summary, these observations identify that expression of TPL-2 in CD11c^+^ cells and Ccl24-dependent pathways can accelerate expulsion of *H*. *polygyrus*.

Upon infection, *H*. *polygyrus* larvae breach the epithelium and mucosa of the small intestine, exposing the host to pathogen-associated molecular patterns (PAMPs), and eliciting anti-microbial responses. As a consequence, changes in microbiota composition have been observed following *H*. *polygyrus* infection [26, 35, 36]. Furthermore, helminth-associated intestinal microbiota alterations have been linked to helminth-mediated immunoregulation in distal sites [37]. Given that TPL-2 is downstream of TLR4 and IL-1R-signaling and that TPL-2-deficent mice are more susceptible to bacterial infection due to reduced TNFα and IL-1β secretions [12], we analyzed the changes in microbiota following infection of WT and *Map3k8*^*–/–*^mice. In accordance with previous studies, we observed changes in the fecal microbiota composition of *H*. *polygyrus* infected WT mice. However, several of these changes were not observed in *Map3k8*^*–/–*^mice (grey portion of **Fig 2B**), suggesting that host TPL-2-dependent pathways contributed to *H*. *polygyrus*-associated alterations in microbiota composition. Specifically, we observed increases in the proportional abundances of *Alphaproteobacteria* spp. and *Clostridia* spp. following infection, which were unchanged in *Map3k8*^*–/–*^mice. Normalizing these differences by co-housing, however, did not impact the accelerated resistance observed in *Map3k8*^*–/–*^mice, indicating that differences in the microbial composition between WT and *Map3k8*^*–/–*^mice were not responsible for resistance to *H*. *polygyrus* infection in *Map3k8*^*–/–*^mice.

Previous studies have demonstrated the role of TPL-2 in preventing severe airway allergy [17, 20] and limiting Th2 mediated immunopathology [18]. To date, there have been no studies investigating the role of TPL-2 in regulating helminthiasis, wherein type-2 responses are protective. Furthermore, mechanisms of resistance to *H*. *polygyrus* infection [3, 4, 6] have been primarily observed in secondary challenge infections post drug treatment. In the present study, we demonstrate a previously unidentified immunoregulatory mechanism with TPL-2 deficiency promoting spontaneous worm expulsion during primary *H*. *polygyrus* chronic infection. Resistance to *H*. *polygyrus* infection in TPL-2 deficient mice was not due to a detectable increase in adaptive type-2 immunity in the spleen, mLN and PP, determined by *Il4*^GFP^ expression from CD4^+^ T cells, IL-5 and IL-13 secretion from HEX stimulated mLN cultures or levels of antigen-specific serum IgG1, all of which have been implicated in mediating protection from *H*. *polygyrus* [5]. This contrasts with the role of TPL-2 in airway allergy and *S*. *mansoni* induced immunopathology where we have previously observed enhanced Th2 responses and increased serum antibody responses in *Map3k8*^*–/–*^mice compared to WT mice [17, 18]. It is likely these differences arise from site and cell specific roles of TPL-2 in regulating inflammatory responses. Our new observations suggest that TPL-2- regulated innate pathways upstream of Th2 cell differentiation, class switch recombination and antibody secretion, orchestrating mechanisms of resistance to intestinal *H*. *polygyrus*. Early transcriptional differences in *Map3k8*^*–/–*^mice pointed towards genes associated with chemotaxis and homing of leukocytes to infected tissue, in addition to genes associated with alternatively activated macrophages / dendritic cells. These genes and gene associated pathways have previously been linked with challenge infections with an influx of neutrophils, alternatively activated macrophages, CD4^+^ T cells and CD11c^+^ dendritic cells in a type-2 granuloma leading to the killing of tissue larvae [34]. Thus it appeared that TPL-2-deficient mice mounted an accelerated ‘memory-like’ type-2 inflammatory response, rather than a primary response.

Accelerated innate pathways may result in more effective killing of invading larvae during a primary infection compared to adaptive immune pathways, that primarily target established adult worms. One such accelerated TPL-2-regulated pathway involved Ccl24. It was recently reported that IL-33 secretion, which is released soon after *H*. *polygyrus* infection [38], can activate group 2 innate lymphoid cells (ILC2) and drive Ccl24 expression at mucosal surfaces [39]. Although we did not detect differences in *Il33* transcripts or differences in the number or the activation states of ILC2 cells, we observed significantly more *Ccl24* in TPL-2-deficient mice. Although the precise mechanisms regulating these upstream events remain unclear, it is possible that early IL-33-ILC2 derived type-2 cytokines could activate TPL-2-deficient cells leading to enhanced production of Ccl24. In support of this activation of myeloid cells, GM-CSF matured dendritic cells and lung resident CD11b^+^ myeloid cells respond to IL-4R signaling to produce Ccl24 [27, 40] and IL-33 and ILC2 expansion is actively inhibited by *H*. *polygyrus*-derived excretory secretory products [41], suggesting that *H*. *polygyrus* may have evolved to directly inhibit this otherwise anthelmintic response.

Using a cell-specific deletion system, we identified that accelerated expulsion of *H*. *polygyrus* tracked with deletion of *Map3k8* in CD11c-expressing, but not *Villin*-, *Cd4*- or *LysM*-expressing cells. We have previously observed that TPL-2-regulated Ccl24 expression in CD11c^+^ dendritic cells following HDM challenge [17]. Here we demonstrate that LP-derived CD11c^+^ CD11b^+^ cells express increased *Ccl24*. Taken together with previous observations, we hypothesized that an early inflammatory model, where IL-33 [38], and/or IL-4 [42] induced Ccl24 secretion by CD11c^+^ cells [27, 43, 44] orchestrates an early inflammatory response. In accordance with this hypothesis elevated Ccl24 was concurrent with an increase in the frequency of eosinophils, neutrophils, monocytes and activated *Il4*^*GFP+*^ Th2 cells in the LP of *H*. *polygyrus* infected *Map3k8*^*–/–*^mice. CCR3 expressing myeloid cells and T lymphocytes can contribute to anthelminthic immunity [1, 31–33]. In support of this, we demonstrate that increased Ccl24 correlated with increased parasite killing in *Map3k8*^*–/–*^mice. In addition to an increased frequency of myeloid cells and *Il4*^*GFP+*^ Th2 cells in *Map3k8*^*–/–*^mice, we observed a significant decrease in the frequency and a trend for a decrease in the number of other lymphocyte subsets including, CD4^+^ T cells, CD8^+^ T cells and CD19^+^ B cells in *Map3k8*^*–/–*^mice. These observations suggest a change in the cellular landscape in the LP of D5 *H*. *polygyrus* infected TPL-2 deficient mice towards a myeloid and Th2 cell-biased environment, which may contribute to an early type-2 ‘memory-like’ inflammatory response.

Ex-vivo TPL-2 deficient CD11c^+^ CD11b^+^ cells expressed significantly increased *Ear11*, *Retnla* and had a trend for increased *Arg1* and *Chil3*, markers associated with type-2 alternatively activated macrophages / dendritic cells. Neutralization of Ccl24 led to a significant reduction in *Arg1*, *Retnla* and *Ear11* and a trend for a decrease in *Chil3*, in *H*. *polygyrus* infected TPL-2 deficient mice. Correlating with the loss of these markers, we observed a loss of resistance to infection in TPL-2 deficient mice. Taken together, an accelerated ‘memory-like’ type-2 responses, mediated by CD11c^+^ cells in *H*. *polygyrus* infected TPL-2 deficient mice, led to an influx of inflammatory myeloid and Th2 cells, via Ccl24, accelerating worm expulsion. These observations point to a previously unidentified role of TPL-2 in restricting Ccl24-mediated immunity to *H*. *polygyrus*.

## Materials and methods

### Ethics statement

All animal experiments were carried out following UK Home Office regulations (project license 80/2506 and 70/8809) and were approved by The Francis Crick Institute Ethical Review Panel.

#### Animals and generation of bone marrow chimeric mice

All mice used in this study were maintained under specific pathogen-free conditions at the Mill Hill Laboratory, The Francis Crick Institute. Strains used included: WT C57BL/6, *Map3k8*^*–/–*^[10] on a C57BL/6 background, *Il4*^*GFP*^*Foxp3*^*RFP*^*10*^*BIT*^ (B6.*Il4*^*GFP*^ crossed with B6.*Foxp3*^*RFP*^ and B6.*10*^*BIT*^), *Map3k8*^*–/–*^*Il4*^*GFP*^*Foxp3*^*RFP*^*10*^*BIT*^ (*Map3k8*^*–/–*^crossed with *Il4*^*GFP*^*Foxp3*^*RFP*^*10*^*BIT*^), *Cd4*^*Cre*^*Map3k8*^*flfl*^*R26*^*eYFP*^ (B6.*Cd4*^*Cre*^ crossed with *Map3k8*^*flfl*^ *and* B6.*R26*^*eYFP*^), *Lysm*^*Cre*^*Map3k8*^*flfl*^*R26*^*eYFP*^ (B6.*Lysm*^*Cre*^ crossed with *Map3k8*^*flfl*^ *and* B6.*R26*^*eYFP*^), *Villin*^*Cre*^*Map3k8*^*flfl*^ (B6.*Villin*^*Cre*^ crossed with *Map3k8*^*flfl*^*) and Cd11c*^*Cre*^*Map3k8*^*flfl*^*R26*^*eYFP*^ (B6.*Cd11c*^*Cre*^ crossed with *Map3k8*^*flfl*^ *and* B6.*R26*^*eYFP*^).

#### *H*. *polygyrus* infection and *in vivo* treatments

Mice were infected with 200 *H*. *polygyrus* L3 larvae by oral gavage. Worms were counted in the lumen at day 14, day 21 or day 28 and wall of the intestine at day 5 following infection. For the *in vivo* blocking of Ccl24, neutralizing antibodies diluted in PBS (Ccl24 antibodies (CCL24/Eotaxin-2 MAb (Clone 106521, R&D systems—Biotechne) and CCL24/Eotaxin-2 Affinity Purified Polyclonal Ab, R&D systems—Biotechne) were mixed and used at 50μg/dose (monoclonal) and 10μg/dose (polyclonal). Isotype controls rat IgG_2A_ (Clone 54447, R&D systems–Biotechne) and normal goat IgG (R&D systems–Biotechne) were mixed and used at a similar dose. The mice were treated with the neutralizing antibodies 1 day before infection with *H*. *polygyrus* and days 1, 3 and 5 post infection. The mice were culled on day 21 for luminal worm counts. For the evaluation of Ccl24 regulated gene expression changes at D5 post infection with *H*. *polygyrus*, the mice were treated with the neutralizing antibodies 1 day before infection with *H*. *polygyrus* and days 1 and 3 post infection.

#### Cell isolation and culture

Spleen, mLN and PP cells were made into a single cell suspension by gently mashing through a 40μM filter (ThermoScientific) and prepared for FACS analysis or sorting. Lamina propria (LP) cells were isolated as described previously [42]. For FACS analysis or sorting cells were resuspended in complete Iscove’s Modified Dulbecco’s Medium (cIMDM) containing 1% fetal bovine serum (FBS), 1mM EDTA, 100U/ml Penicillin (Gibco) and 100μg/ml Streptomycin (Gibco), 8mM L-glutamine (Gibco) and 0.05mM 2-mercaptoethanol (Gibco). For HEX specific cell cultures, mLN cell suspensions were cultured in cIMDM containing 10% FBS, 100U/ml Penicillin and 100μg/ml Streptomycin, 8mM L-glutamine and 0.05mM 2-mercaptoethanol in the presence of HEX (10μg/ml) for 3–4 days and supernatants were harvested for ELISAs.

#### RT- PCR and ELISAs

Cell pellets were lysed in Buffer RA1 (Macherey-Nagel). Total RNA was isolated as per manufacturer’s protocol for RNA isolation from small samples. Total RNA was extracted from small intestinal duodenal tissue preserved in RNAlater (Sigma) using Qiazol homogenization and the RNeasy mini kit (Qiagen). Total RNA was reverse transcribed using the QuantiTect Reverse Transcription Kit (Qiagen). cDNA produced was used for real time quantitative PCR with Power SyBrGreen (Applied Biosystems). The expression level for *Ccl24* (For: GGGTCATCTTCATCACCAAGAAGG; Rev: GGTCTGTCAAACCCCAAAGCA), *Ear11* (FOR: CTTCCCAGTGGTTTGACATCC; Rev: CCCGTCTTTGCAGATCTTACG) was normalized to hypoxanthine-guanine phosphoribosyl transferase (*Hprt*) expression and reported. Primer sequences for *Arg1*, *Retnla*, *Chil3* and *Hprt* have been previously reported [42]. IL-5 and IL-13 were measured using DuoSet ELISA kits, according to the manufacturer’s instructions (R&D systems—Biotechne). For antigen specific IgG1 ELISAs, 96 well plates were coated overnight with *H*. *polygyrus* adult worm antigen mix (HEX) at the concentration 5μg/ml. The plates were blocked and incubated with serum in serial dilutions overnight. The plates were washed and incubated in biotin Rat anti-mouse IgG1 (Invitrogen). The samples were detected with Streptavidin HRP (BD Pharmingen) and ABTS One Component HRP Microwell Substrate (SurModics).

#### Flow cytometry

Flow cytometry analysis was performed using a LSR II and the LSRFortessa X-20 (BD Biosciences) analysers. For analysis, single cell suspensions resuspended in cIMDM. The cells were stained with antibodies in PBS containing 2% FBS for 25 min at 4°C, washed and fixed in 2–4% paraformaldehyde for FACS analysis. For all analysis, viability of the cells was determined using the LIVE/DEAD Fixable Blue kit (Life Technologies). Antibodies used were purchased with eBioscience, Biolegend or BD Pharmingen. They include: CD3 (17A2), CD4 (RM4-5, GK1.5), CD11b (M1/70), CD11c (N418), CD19 (6D5, eBio1D3), CD25 (PC61), CD44 (IM7), CD45 (30-F11), CD49b (DX5), F4/80 (BM8), I-A/I-E (M5/114.15.2), KLRG1 (2F1), Ly6G (1A8), NK1.1 (PK136), Sca1 (E13 161.7), SiglecF (E50-2440), TCR β chain (H57-597), TCRγδ (GL3), Ter119 (TER-119), Thy1.2 (53 2.1). Lineage panel for the ILC2 staining was defined as CD4^+^/ CD8^+^/ CD3^+^/ CD19^+^/ TCRβ^+^/ TCRγδ^+^/ NK1.1^+^/ CD49b^+^/ CD11c^+^/ CD11b^+^/ Gr-1^+^/ Ter119^+^. Staining was performed in the presence of FcR Blocking Reagent (Miltenyi Biotec).

#### ATP assay for adult *H*. *polygyrus* worms

Adult *H*. *polygyrus* ATP was measured using the CellTiter-Glo Luminescent Cell Viability Assay (Promega). Briefly, *H*. *polygyrus* adult worms were removed from the duodenum of mice and homogenised using a motorised pestle in 110 μL of PBS and 110 μL of CellTiter-Glo Reagent. The homogenate was incubated for 10 minutes at room temperature before centrifugation at 1000g for 3 minutes. 200 μL of the supernatant was transferred to a 96 well opaque-walled plate and incubated for 10 minutes at room temperature before recording luminescence. An ATP standard curve was generated by using recombinant ATP (Promega) as detailed in the CellTiter-Glo Luminescent Cell Viability Assay instructions.

#### Fecal DNA extraction and 454 sequencing

Fecal pellets were homogenized and DNA was extracted using the FastDNA Spin Kit for Soil (MP Biomedicals) as per the manufacturer’s recommendations. 16S rRNA genes were PCR amplified from these DNA extractions, sequenced and analyzed largely as described previously [45]. However, for the present study, reads shorter than 300 bp in length after quality filtering were discarded, and each sample was rarefied to a sequence depth of 1121 sequence reads per sample prior to further analysis. All diversity and compositional comparisons were carried out at this sequence depth. Sequence data have been deposited at the European Nucleotide Archive under Study Accession Number ERP005257 and/or Sample Accession Number ERS421609. The barcodes used for each of the samples are listed in **S1 Table**.

#### Microarray analysis

WT and *Map3k8*^*–/–*^intestinal tissue were isolated from *H*. *polygyrus* infected mice day 5 post-infection. The tissue samples were homogenized in QIAzol (Qiagen) and total RNA was isolated from tissues and cells using RNAeasy mini-spin columns (Qiagen) according to the manufacturer’s instructions. cDNA was generated from 5 ng of total RNA using the WT-Ovation Pico system (version 1) RNA Amplification System followed by double-stranded cDNA synthesis using the WT-Ovation Exon Module. cDNA quality was determined by the Systems Biology Unit at The Francis Crick Institute using an Agilent BioAnalyzer and through hybridization performance on Affymetrix GeneChip mouse Genome 430A 2.0 microarray (Affymetrix). Microarray data were quantile-normalized and analyzed using GeneSpring software (Agilent). Differentially expressed genes were determined using ANOVA and *t* tests. Genes with false discovery rate-corrected P values <0.05 and fold change values ≥2.0 were considered significant. 3 biological replicates of each sample were used.

#### Statistical analysis

Data sets were compared by Mann Whitney test using GraphPad Prism (V.5.0). Differences were considered significant at p ≤ 0.05.

## Supporting information

S1 Table Barcode details for each sample. A list of barcodes used for each of the fecal DNA samples, as described in Materials and Methods.
(XLSX)

S1 Fig *H*. *polygyrus* infection in *Map3k8*^*–/–*^mice at D5 and D7. A) Frequency of CD4^+^ *Il4*^GFP+^ cells in the spleen, mLN and PP of D5 *H*. *polygyrus* infected WT and *Map3k8*^*–/–*^mice. B) Frequency of CD4^+^ *FoxP3*^RFP+^ cells in the spleen, mLN and PP of D5 *H*. *polygyrus* infected WT and *Map3k8*^*–/–*^mice. C) Total cell counts in the spleen, mLN and PP of D5 and *H*. *polygyrus* infected WT and *Map3k8*^*–/–*^mice. D) Frequency of CD4^+^ TCRβ^+^ CD44hi *Il4*^GFP+^ cells in the spleen, mLN and PP of D7 *H*. *polygyrus* infected WT and *Map3k8*^*–/–*^mice. E) Frequency of CD4^+^ TCRβ^+^ *Foxp3*^RFP+^ cells in the spleen, mLN and PP of D7 *H*. *polygyrus* infected WT and *Map3k8*^*–/–*^mice. F) Total cell counts in the spleen, mLN and PP of D7 *H*. *polygyrus* infected WT and *Map3k8*^*–/–*^mice. G) HEX-specific IL-5 and IL-13 in mLN cell culture supernatants of D7 *H*. *polygyrus* infected WT and *Map3k8*^*–/–*^mice. H) Worm burden in the duodenal wall of WT and *Map3k8*^*–/–*^mice at D5 post infection with 200 L3 stage *H*. *polygyrus* larvae. Data is representative of 2 independent experiments with 3–4 mice/genotype. * denotes p≤0.05 using Mann-Whitney test.
(TIF)

S2 Fig Shannon diversity index of fecal microbiota in *H*. *polygyrus* infected WT and *Map3k8*^*–/–*^mice. A) Shannon diversity index calculation was performed using the mothur software package [46] and average indices were plotted for the different genotypes within the two groups of mice over the course of the experiment. Data represents 5 mice/genotype. * denotes p≤0.05 using unpaired two-tailed t test test.
(TIF)

S3 Fig Gene expression and cellular changes in D5 *H*. *polygyrus* infected WT and *Map3k8*^*–/–*^mice. A-C) mRNA expression of (F) *Retnla*, (G) *Arg1 and* (H) *Chil3* in *ex vivo* CD11c+ CD11b+ cells from the small intestinal LP of D5 *H*. *polygyrus* infected WT and *Map3k8*^*–/–*^mice. D-H) Frequency and number of (D) total CD4^+^ T cells (live/CD45^+^/CD4^+^/CD3^+^/TCRβ^+^); (E) CD8^+^ T cells (live/CD45^+^/CD4^+^/CD3^+^/TCRβ^+^); (F) B cells (live/CD45^+^/CD4^-^/CD8^-^/CD19^+^); (G) Macrophages (live/CD45^+^/SiglecF-/CD11b^+^/Ly6G^-^/F4/80^+^); (H) ILC2s (live/CD45^+^/Lineage^-^/Thy1.2^+^/KLRG1^+^/Sca1^+^) in the intestinal LP of D5 *H*. *polygyrus* infected WT and *Map3k8*^*–/–*^mice. Data from A-C) is representative of a single experiment with 3 biological replicates with each replicate consisting of 3 mice/replicate/group. Data from D-H) is representative of 2 independent experiments with 3–6 mice/group. * denotes p≤0.05 using Mann-Whitney test.
(TIF)
