# Supplementary figures and images for: TPL-2 restricts Ccl24-dependent immunity to Heligmosomoides polygyrus
Source: PLoS Pathog. 2017 Jul 31;13(7):e1006536. doi: 10.1371/journal.ppat.1006536 (PMC5560741; doi:10.1371/journal.ppat.1006536)

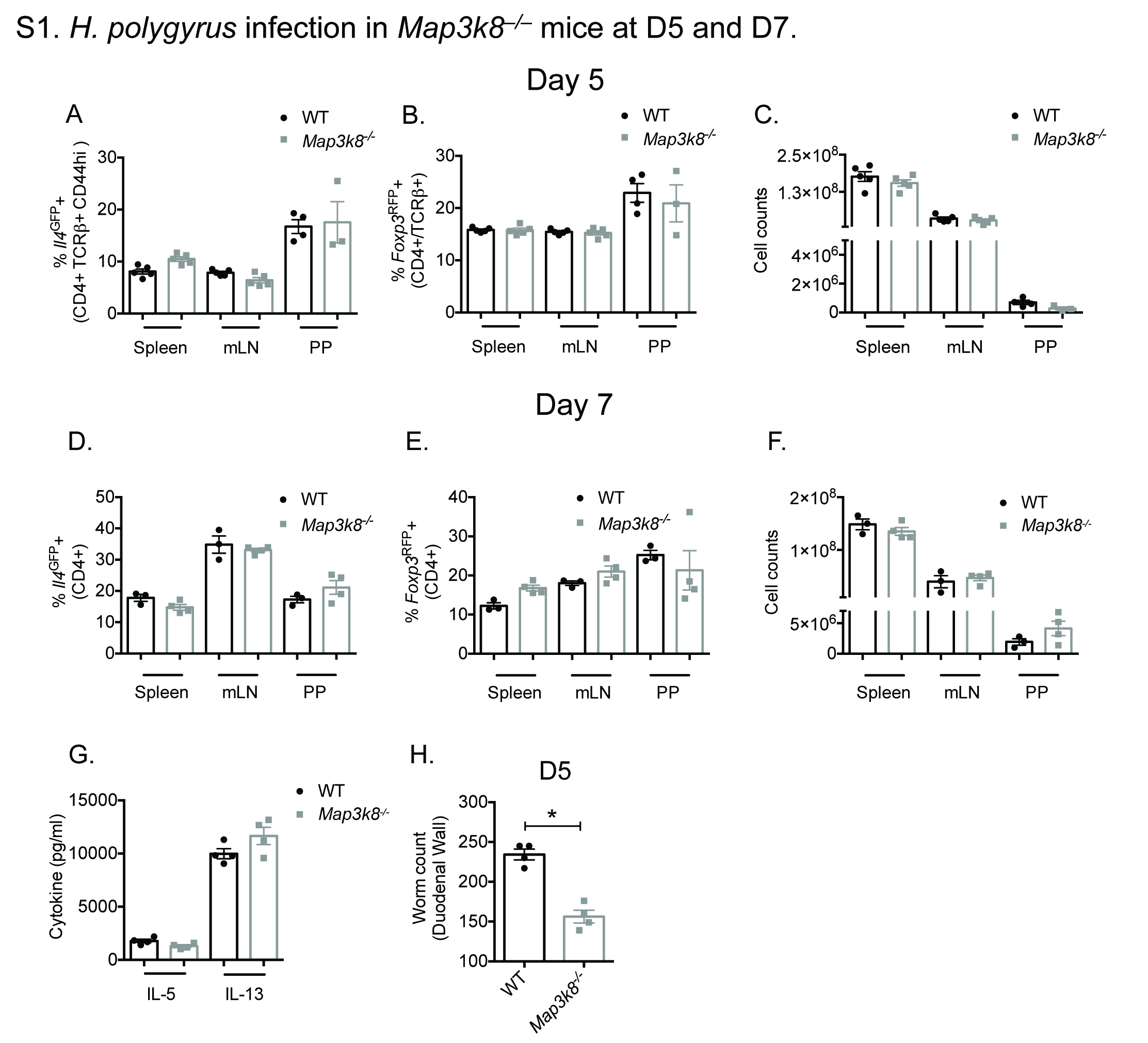

Supplement: S1 Fig — A) Frequency of CD4+ Il4GFP+ cells in the spleen, mLN and PP of D5 H. polygyrus infected WT and Map3k8–/–mice. B) Frequency of CD4+ FoxP3RFP+ cells in the spleen, mLN and PP of D5 H. polygyrus infected WT and Map3k8–/–mice. C) Total cell counts in the spleen, mLN and PP of D5 and H. polygyrus infected WT and Map3k8–/–mice. D) Frequency of CD4+ TCRβ+ CD44hi Il4GFP+ cells in the spleen, mLN and PP of D7 H. polygyrus infected WT and Map3k8–/–mice. E) Frequency of CD4+ TCRβ+ Foxp3RFP+ cells in the spleen, mLN and PP of D7 H. polygyrus infected WT and Map3k8–/–mice. F) Total cell counts in the spleen, mLN and PP of D7 H. polygyrus infected WT and Map3k8–/–mice. G) HEX-specific IL-5 and IL-13 in mLN cell culture supernatants of D7 H. polygyrus infected WT and Map3k8–/–mice. H) Worm burden in the duodenal wall of WT and Map3k8–/–mice at D5 post infection with 200 L3 stage H. polygyrus larvae. Data is representative of 2 independent experiments with 3–4 mice/genotype. * denotes p≤0.05 using Mann-Whitney test. (TIF) [file ppat.1006536.s002.tif]

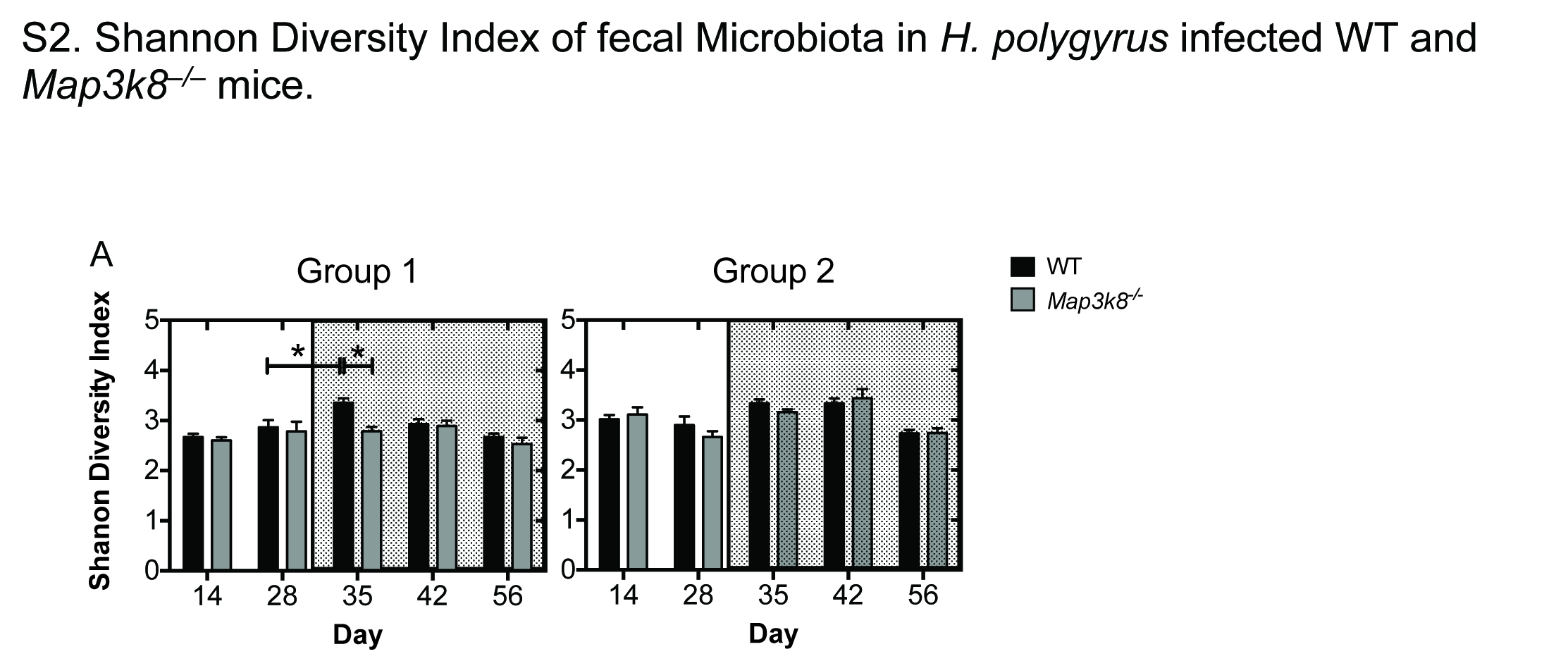

Supplement: S2 Fig — A) Shannon diversity index calculation was performed using the mothur software package [46] and average indices were plotted for the different genotypes within the two groups of mice over the course of the experiment. Data represents 5 mice/genotype. * denotes p≤0.05 using unpaired two-tailed t test test. (TIF) [file ppat.1006536.s003.tif]

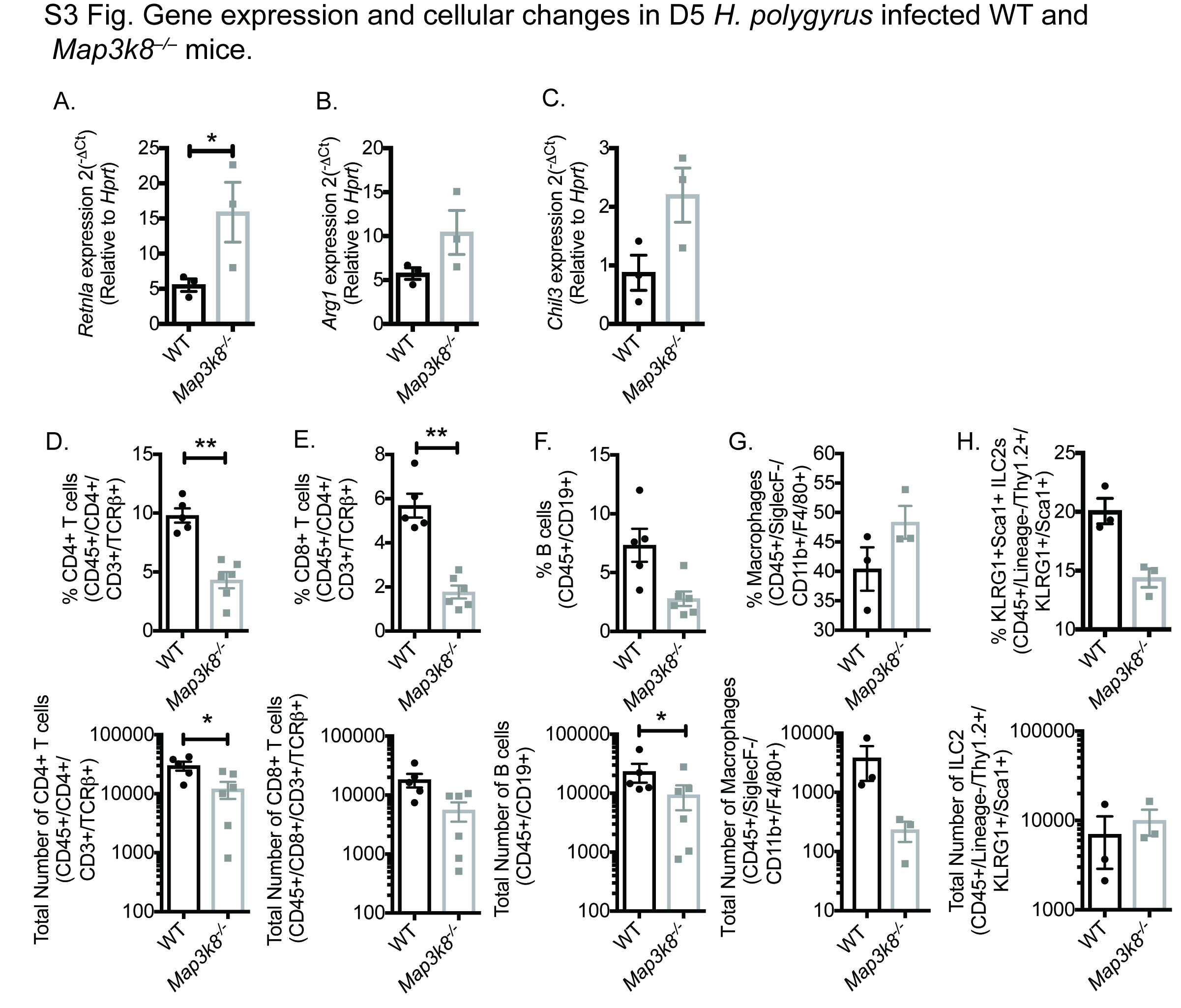

Supplement: S3 Fig — A-C) mRNA expression of (F) Retnla, (G) Arg1 and (H) Chil3 in ex vivo CD11c+ CD11b+ cells from the small intestinal LP of D5 H. polygyrus infected WT and Map3k8–/–mice. D-H) Frequency and number of (D) total CD4+ T cells (live/CD45+/CD4+/CD3+/TCRβ+); (E) CD8+ T cells (live/CD45+/CD4+/CD3+/TCRβ+); (F) B cells (live/CD45+/CD4-/CD8-/CD19+); (G) Macrophages (live/CD45+/SiglecF-/CD11b+/Ly6G-/F4/80+); (H) ILC2s (live/CD45+/Lineage-/Thy1.2+/KLRG1+/Sca1+) in the intestinal LP of D5 H. polygyrus infected WT and Map3k8–/–mice. Data from A-C) is representative of a single experiment with 3 biological replicates with each replicate consisting of 3 mice/replicate/group. Data from D-H) is representative of 2 independent experiments with 3–6 mice/group. * denotes p≤0.05 using Mann-Whitney test. (TIF) [file ppat.1006536.s004.tif]
